# Risk factors for radiation-induced lung injury in patients with advanced non-small cell lung cancer: implication for treatment strategies

**DOI:** 10.1186/s12957-021-02321-3

**Published:** 2021-07-16

**Authors:** Sha Sha, Jigang Dong, Maoyu Wang, Ziyu Chen, Peng Gao

**Affiliations:** Department of Radiotherapy, Jiaozhou Central Hospital, No. 29 Xuzhou Road, Jiaozhou City, Qingdao, 266300 China

**Keywords:** Radiation, Lung injury, Non-small cell lung cancer, Treatment, Care

## Abstract

**Background:**

The radiation-induced lung injury (RILI) in patients with advanced non-small cell lung cancer (NSCLS) is very common in clinical settings; we aimed to evaluate the risk factors of RILI in NSCLS patients, to provide insights into the treatment of NSCLS.

**Methods:**

NSCLC patients undergoing three-dimensional conformal radiotherapy (3D-CRT) in our hospital from June 1, 2018, to June 30, 2020, were included. The characteristics and treatments of RILI and non-RILI patients were analyzed. Logistic regression analyses were conducted to assess the risk factors of RILI in patients with NSCLC.

**Results:**

A total of 126 NSCLC patients were included; the incidence of RILI in NSCLC patients was 35.71%. There were significant differences in diabetes, smoke, chronic obstructive pulmonary disease (COPD), concurrent chemotherapy, radiotherapy dose, and planning target volume (PTV) between the RILI group and the non-RILI group (all *P* < 0.05). Logistic regression analyses indicated that diabetes (OR 3.076, 95%CI 1.442~5.304), smoke (OR 2.745, 95%CI 1.288~4.613), COPD (OR 3.949, 95%CI 1.067~5.733), concurrent chemotherapy (OR 2.072, 95%CI 1.121~3.498), radiotherapy dose ≥ 60 Gy (OR 3.841, 95%CI 1.932~5.362), and PTV ≥ 396 (OR 1.247, 95%CI 1.107~1.746) were the independent risk factors of RILI in patients with NSCLC (all *P* < 0.05).

**Conclusions:**

RILI is commonly seen in NSCLS patients; early targeted measures are warranted for patients with those risk factors; future studies with larger sample sizes and different areas are needed to further elucidate the influencing factors of RILI in the treatment of NSCLS.

## Background

Non-small cell lung cancer (NSCLC) is a common type of clinical lung cancer, which accounts for about about 40% of the total number of lung cancers [1, 2]. Local radiotherapy treatment is an important treatment of NSCLC, and radiation-induced lung injury (RILI) is a common complication of thoracic radiation therapy. Once RILI occurs, it is often irreversible and significant [3]. Therefore, the early prevention and treatment of RILI are crucial to improve the prognosis of patients.

Three-dimensional conformal radiotherapy (3D-CRT) refers to multiple beam irradiation technology that uses a series of different weights, different field shapes, and sizes to irradiate the target area from different directions [4–6]. A conformal lead that is consistent with the shape of the lesion is used to make the high-dose area consistent with the shape of the target area in three-dimensional space and at the same time reduce the dose of normal tissues around the target area [7, 8]. RILI is one of the main side effects of thoracic cancer radiotherapy. The clinical incidence of RILI is reported in previous studies to be 5.12 to 36.07%, which can significantly affect the treatment and prognosis of patients [9–11]. Although 3D-CRT can reduce radiation damage to normal tissues through precise quantitative control of cancer radiation dose, the occurrence of RILI with clinical symptoms is still very common [12]. RILI patients have a series of non-specific respiratory symptoms due to severe lung injury, which will seriously affect the patient’s quality of life [13–15]. How to predict and prevent the occurrence of RILI has become an urgent problem to be solved in radiotherapy [16]. Therefore, we aimed to evaluate the risk factors of RILI in NSCLC patients, to provide insights into the clinical prevention and treatment of RILI.

## Methods

### Ethical considerations

In this study, all methods were performed in accordance with the relevant guidelines and regulations. This present study had been checked and approved by the ethical committee of our hospital (approval number: DE180042), all the participants had been well-informed, and written informed consents had been obtained from all the included patients.

### Patients

This study selected NSCLC patients who underwent 3D-CRT in our hospital from June 1, 2018, to June 30, 2020, as study populations. The inclusion criteria of the patients were as follows: (1) patients were diagnosed with pathological examination as NSCLC [17, 18]; (2) age< 70 years old, (3) Karnofsky score ≥ 70 points, (4) patients received 3D-CRT, and (5) the follow-up period after radiotherapy was ≥ 6 months. The exclusion criteria for patients were as follows: (1) distant metastasis occurred during radiotherapy, (2) there were interruptions during radiotherapy > 1 week, (3) surgery treatment was performed during radiotherapy, and (4) patients were unwilling to participant in this present study.

### 3D-CRT

Gamma MEDx 100 (German) was used for the 3D-CRT. The patient was fixed with hydrolyzed plastic forming technology, and the arms are raised and crossed on the top of the forehead. The patient kept breathing calmly and performed a spiral CT scan, ranging from the midline of the neck to 3 cm below the diaphragm, including the whole lung tissue, with a thickness of 3 mm. The positioning image was obtained, the Z line value was approved, and the X, Y, and Z coordinates of the marked points on the patient’s body surface. For the parameters and repeated positioning readings, we used bony markers that are not easy to move as the marking points on the body surface and transmitting the positioning images obtained from the positioning scan to the Curer 8.0 treatment planning system via the network. Target area delineation: we delineated the gross tumor volume (GTV) layer by layer under the lung window of the CT image after the 3D reconstruction, and the clinical target volume (CTV) was obtained by placing 6 mm of squamous cell carcinoma GTV. For adenocarcinoma, GTV was placed 8 mm to obtain CTV, and CTV was expanded by 5 mm to obtain the planned target volume (PTV). If the patient’s lung function was poor or the CTV is large, the CTV should be manually modified according to the patient’s lung function status and breathing amplitude. We appropriately increased the scope of the patient’s upper and lower PTV extension up to 10~15 mm outside the CTV. The radiation therapy dose was 46~70 Gy (the median dose is 60 Gy), 5 times per week, 1.8~2.0 Gy per time. Normal tissue dose limits: spinal cord D_max_ ≤ 45 Gy, esophagus D_max_ ≤ 60 Gy, lung V_5_ ≤ 65%, lung V_20_ ≤ 25%, lung V_30_ ≤ 20%.

### Chemotherapy

The chemotherapy regimen used in our study was as follows: paclitaxel (Xinwei, Shanghai, S0190335) or docetaxel (Gynesier, German, C901241511) + cisplatin (Minhua, Shenzhen, W291101) or carboplatin (Huangchen, Zhengzhou, 1910418). The first-line regimen of intravenous infusion of paclitaxel (135~175 mg m^−2^) (day 1), docetaxel (65~75 mg m^−2^) (day 1), cisplatin (80–100 mg m^−2^) (days 2 and 3), and carboplatin (300–350 mg m^−2^) (day 2). At 21–28 days for 1 cycle, a total of 2 to 6 cycles were completed.

### Diagnostic criteria of RILI

RILI toxicity was assessed with the Common Terminology Criteria for Adverse Events version 4.0, which was divided into 0 to 5 levels: grade 0—no obvious symptoms or signs change before and after treatment; grade 1—mild cough or shortness of breath after radiotherapy; grade 2—cough was persistent and requires medicine to relieve it and asthma after exercise; grade 3: severe cough, asthma, and shortness of breath were presented in the resting state, which could not be relieved by drugs, and the imaging examination revealed radiation pneumonia. Glucocorticoid therapy was effective; grade 4—dyspnea, oxygen, or assisted ventilation; and grade 5—patient death due to ventilatory failure and lung failure.

### Data collection

Two authors collected the following personal characteristics and treatment data of patients: gender, age, body mass index (BMI), diabetes, hypertension, hyperlipidemia, smoke, chronic obstructive pulmonary disease (COPD), pathological type, concurrent chemotherapy, Karnofsky percent score (KPS), tumor staging, cancer size, radiotherapy dose (Gy), V_5_, V_10_, V_20_, V_30_, MLD, GTV, and PTV.

### Statistical analysis

We used the SPSS 24.0 statistical software for statistical analysis. Enumeration data was expressed as cases and percentages, and the chi-square test was used for comparison between the groups; measurement data was expressed as mean ± standard deviation, and the t test was used for comparison between the groups. Logistic regression analyses were conducted to assess the risk factors of RILI in patients with NSCLC. *P* < 0.05 indicates that the difference was statistically significant.

## Results

### The characteristics of included patients

A total of 126 NSCLC patients were included, of whom 45 patients had RILI; the incidence of RILI in NSCLC patients was 35.71%. As presented in Table 1, there were significant differences in diabetes, smoke, COPD, concurrent chemotherapy, radiotherapy dose, and PTV between the RILI group and non-RILI group (all *P* < 0.05). But no significant differences in gender, age, BMI, hypertension, hyperlipidemia, pathological type, KPS, tumor staging, cancer size, V_5_, V_10_, V_20_, V_30_, MLD, and GTV between the RILI group and the non-RILI group were found (all *P* > 0.05).

**Table 1:** The characteristics of included patients

Variables	RILI group (*n* = 45)	Non-RILI group (*n* = 81)	χ^2^/t	P
Male/female	31/14	48/33	1.029	0.112
Age (years)	61.01 ± 8.52	60.17 ± 9.33	4.381	0.065
BMI (kg/m^2^)	22.27 ± 4.15	22.09 ± 3.98	3.424	0.082
Diabetes	24 (53.33%)	20 (24.69%)	1.187	0.013
Hypertension	25 (55.56%)	44 (54.32%)	1.272	0.104
Hyperlipidemia	10 (22.22%)	16 (19.75%)	1.489	0.076
Smoke	31 (68.89%)	24 (29.63%)	1.233	0.014
COPD	18 (40%)	22 (27.16%)	1.504	0.046
Pathological type				
Squamous cell carcinoma	32 (71.11%)	58 (71.61%)	1.127	0.088
Adenocarcinoma	13 (28.89%)	23 (28.39%)		
Concurrent chemotherapy	39 (86.67%)	61 (75.31%)	1.231	0.039
KPS	84.43 ± 12.72	82.76 ± 11.23	1.107	0.092
Tumor staging				
IIIa	24 (53.33%)	44 (54.32%)	1.145	0.106
IIIb	22 (48.89%)	37 (45.68%)		
Cancer size (cm)	5.88 ± 3.26	5.65 ± 2.64	1.230	0.085
Radiotherapy dose (Gy)	63.75 ± 11.32	52.48 ± 10.66	9.277	0.001
V_5_	54.44 ± 11.68	53.26 ± 12.42	11.207	0.073
V_10_	32.76 ± 8.01	29.19 ± 9.54	8.021	0.081
V_20_	22.12 ± 5.37	21.35 ± 5.95	9.101	0.089
V_30_	12.31 ± 4.98	11.93 ± 4.17	4.116	0.105
MLD	949.59 ± 242.83	898.91 ± 282.44	77.174	0.059
GTV	169.05 ± 52.18	149.77 ± 61.72	18.101	0.091
PTV	472.34 ± 69.64	321.58 ± 79.84	53.374	0.011

*Note*: The percentage of the lung volume that received more than a certain dose of the total lung volume was recorded as V5 (≤ 65%), V10 (≤ 20%.), V20 (≤ 25%), and V30 (≤ 20%) accordingly

*RILI* radiation-induced lung injury, *BMI* body mass index, *COPD* chronic obstructive pulmonary disease, *MLD* mean lung dose, *GTV* gross target volume, *PTV* planning target volume

### Logistic regression analysis on the risk factors of RILI in patients with NSCLC

The variable assignments of multivariate logistic regression were presented in Table 2. As indicated in Table 3, diabetes (OR 3.076, 95%CI 1.442~5.304), smoke (OR 2.745, 95%CI 1.288~4.613), COPD (OR 3.949, 95%CI 1.067~5.733), concurrent chemotherapy (OR 2.072, 95%CI 1.121~3.498), radiotherapy dose ≥ 60 Gy (OR 3.841, 95%CI 1.932~5.362), and PTV ≥ 396 (OR 1.247, 95%CI 1.107~1.746) were the independent risk factors of RILI in patients with NSCLC (all *P* < 0.05).

**Table 2:** The variable assignment of multivariate logistic regression

Factors	Variables	Assignment
RILI	Y	Yes = 1, no = 2
Diabetes	X_1_	Yes = 1, No = 2
Smoke	X_2_	Yes = 1, No = 2
COPD	X_3_	Yes = 1, No = 2
Concurrent chemotherapy	X_4_	Yes = 1, No = 2
Radiotherapy dose (Gy)	X_5_	≥ 56 = 1, < 56 = 2
PTV	X_6_	≥ 396 = 1, < 396 = 2

*RILI* radiation-induced lung injury, *COPD* chronic obstructive pulmonary disease, *PTV* planning target volume

**Table 3:** The logistic regression analysis on the risk factors of RILI in patients with NSCLC

Variables	β	S‾x	OR	95%CI	p
Diabetes	0.173	0.227	3.076	1.442~5.304	0.019
Smoke	0.129	0.214	2.745	1.288~4.613	0.033
COPD	0.103	0.151	3.949	1.067~5.733	0.007
Concurrent chemotherapy	0.124	0.139	2.072	1.121~3.498	0.038
Radiotherapy dose ≥ 60 Gy	0.191	0.122	3.841	1.932~5.362	0.025
PTV ≥ 396	0.131	0.107	1.247	1.107~1.746	0.042

*COPD* chronic obstructive pulmonary disease, *PTV* planning target volume

## Discussions

RILI is one of the important toxic reactions of radiotherapy in the treatment of thoracic tumors. RILI often appears during radiotherapy to 3 months after radiotherapy [19, 20]. It usually manifests as dry cough, fever, shortness of breath, etc. In severe cases, severe breathing difficulties, respiratory failure, and even death can occur. Numerous studies [21–24] have shown that clinical factors, therapeutic factors, metrological factors, and biological factors are closely related to the occurrence of RILI. The results of this study have shown that diabetes, smoke, COPD, concurrent chemotherapy, radiotherapy dose ≥ 60 Gy, and PTV ≥ 396 were the independent risk factors of RILI in patients with NSCLC; special attentions and early interventions are needed for NSCLC patients with those risk factors.

In this study, the incidence of RILI in NSCLC patients that received 3D-CRT was 35.71%, which is consistent with several previous studies [25, 26]. It has been found that the incidence of RILI in patients receiving concurrent chemotherapy is 63.17%, while the incidence of RILI in patients who did not undergo chemotherapy is only 16%, and the incidence of RILI is higher in patients who received paclitaxel + carboplatin [27–30]. We have found that whether to receive concurrent chemotherapy is significantly related to the incidence of RILI, which may be associated with the fact that patients undergoing chemotherapy treatment are prone to lower immune levels and higher organ sensitivity. It has been found that MLD and the percentage of the total lung volume (lung V_5_, V_10_, V_20_, V_30_, V_40_, V_50_, and V_60_) of the lung volume that received more than a certain dose of irradiation are independent risk factors for RP after 3D-CRT treatment of NSCLC [31–33]. Besides, it has been reported that MLD, V_20_, V_30_, V_40_, and V_50_ have predictive significance for RILI in NSCLC patients treated with radiotherapy and chemotherapy [34].

Diabetes, smoking, and COPD are risk factors related to RILI. This study shows that smoking is a risk factor for RILI. Some studies [14, 35, 36] have shown that smoking is an independent protective factor for RP. It is considered that the hypoxia and immunosuppression caused by smoking may be related to the increased lung tolerance of smoking patients [37]. Studies [30, 38] have pointed out that factors such as smoking, COPD, and ventilatory dysfunction can increase the risk of RILI. It is recommended that lung diffusion function be tested before radiotherapy to evaluate lung function status [39]. Therefore, in radiotherapy, attention should be paid to the exposure volume and exposure dose [40]. At the same time, if the patient has diabetes, smoking, COPD, concurrent chemotherapy, and radiotherapy, the total dose should be optimized and preventive measures should be taken to reduce the occurrence of RILI [41]. Previous studies [42, 43] have shown that complications such as chronic obstructive pulmonary disease and ventilatory dysfunction caused by smoking can increase the risk of lung injury. It is recommended that the lung diffusion function be tested before radiotherapy to evaluate the status of lung function.

This study also has certain shortcomings that must be considered. Firstly, the sample size of this study, especially the number of cases in the RILI group, was small, so the statistical efficiency might be underpowered to detect the potential difference. Secondly, given the limited data, we could not do further subgroup analysis on the severity of RILI. Thirdly, radiation-induced lung injuries can also appear after 3 months, limited by collected data; we only analyzed the radiation-induced lung injuries within 3 months; future studies with longer follow-up are needed. Besides, there may be certain differences in radiotherapy doses and schedules in various hospitals, and future large-sample multi-center studies are needed to further analyze and explore related influencing factors of RILI in NSCLC patients.

## Conclusions

In conclusion, we have found that the incidence of RILI in NSCLC patients is 35.71%, and for patients with diabetes, smoke, COPD, concurrent chemotherapy, radiotherapy dose≥60Gy, and PTV ≥ 396, they may have higher risk factors of RILI; early alert and preventative measures are needed for those patients. At present, the prevention and treatment of RILI are still a difficult problem to solve, and its occurrence is related to many factors. It must be noted that our cohort may be exceedingly small heterogenous that cannot be applied to a larger cohort limited by the small sample size. The clinical value of the observation indicators in this study needs to be further verified in future studies. Moreover, the mechanism of RILI and related influencing factors also needs to be explored in the future.

## Data Availability

All data generated or analyzed during this study are included in this published article.

## Abbreviations

RILI: Radiation-induced lung injury
NSCLS: Non-small cell lung cancer
COPD: Chronic obstructive pulmonary disease
PTV: Planning target volume
3D-CRT: Three-dimensional conformal radiotherapy
GTV: Gross tumor volume
CTV: Clinical target volume
BMI: Body mass index
KPS: Karnofsky percent score
